# Higher mid-term revision rates of posterior stabilized compared with cruciate retaining total knee arthroplasties: 133,841 cemented arthroplasties for osteoarthritis in the Netherlands in 2007–2016

**DOI:** 10.1080/17453674.2018.1518570

**Published:** 2018-10-23

**Authors:** Anneke Spekenbrink-Spooren, Liza N Van Steenbergen, Geke A W Denissen, Bart A Swierstra, Rudolf W Poolman, Rob G H H Nelissen

**Affiliations:** 1 Dutch Arthroplasty Register (Landelijke Registratie Orthopedische Implantaten), ’s-Hertogenbosch;; 2 Department of Orthopaedic Surgery, Sint Maartenskliniek, Nijmegen;; 3 Department of Orthopaedic Surgery, Joint Research, OLVGAmsterdam;; 4 Department of Orthopaedic Surgery, Leiden University Medical Center, Leiden, The Netherlands

## Abstract

Background and purpose — The preference for a cruciate retaining (CR) or posterior stabilized (PS) TKA (total knee arthroplasty) system varies greatly between Dutch hospitals, independent of patient characteristics. We examined mid-term revision rates for men and women of different age categories.

Patients and methods — We included all 133,841 cemented fixed-bearing primary CR or PS TKAs for osteoarthritis reported in the Dutch Arthroplasty Register (LROI) in 2007–2016. Revision procedures were defined as minor when only insert and/or patella were revised and as major when fixed components (tibia and femur) were revised or removed. 8-year all-cause revision rates of CR and PS TKAs were calculated using competing-risk analyses. Cox-regression analyses were performed, adjusted for age at surgery, sex, ASA -score, and previous operations.

Results — PS TKAs were 1.5 (95% CI 1.4–1.6) times more likely to be revised within 8 years of the primary procedure, compared with CR TKAs. When stratified for sex and age category, 8-year revision rate of PS TKAs in men <60 years was 13% (CI 11–15), compared to 7.2% (CI 6.1–8.5) of CR TKAs. Less prominent differences were found in older men and women. For men <60 years differences were found for minor (CR 1.8% (CI 1.4-2.5); PS 3.7% (CI 3.0–4.7)) and major revisions (CR 4.2% (CI 3.3–5.3); PS 7.0% (CI 5.6–8.7)).

Interpretation — Patients who received a cemented fixed-bearing primary PS TKA for osteoarthritis are more likely to undergo either a minor or a major revision within 8 years. This is especially prominent for younger men.

In the Netherlands the number of TKAs increased from over 21,000 in 2012 to almost 25,000 in 2016 (LROI 2017). After 8 years, close to 95% of primary TKAs in the Netherlands are still not revised (LROI 2017).

The vast majority of TKA systems are either cruciate retaining (CR) or posterior stabilized (PS) designs. The proportion of PS TKA systems has increased over the years in the Netherlands without any scientific explanation. Theoretically, in patients with a non-functional posterior cruciate ligament (PCL), a PS TKA system has to be used. In patients with a functional PCL, the decision on which design should be used depends largely on the preference and training of the surgeon (Jacobs et al. 2005, van den Boom et al. 2009). In the literature, there is no consensus regarding the outcome of CR compared with PS TKAs. Several studies showed no differences (either for survival, or for functional, clinical, and radiological outcome parameters) for CR compared with PS TKA systems (Jacobs et al. 2005, Verra et al. 2015, Jasper et al. 2016). Other studies concluded that CR TKAs showed better survival rates (Abdel et al. 2011, Vertullo et al. 2017). Data from registries with high completeness and validity (van Steenbergen et al. 2015, LROI 2017) could be valuable when they have valid classification of the type of design (i.e., CR or PS). We evaluated the 8-year revision rates for all CR and PS TKA systems in the Dutch Arthroplasty Register, to determine the association between patient characteristics and revision rates, as well as the characteristics of these revision procedures. Furthermore, an analysis of the reason for revision is performed.

## Patients and methods

### Dutch Arthroplasty Register

The Dutch Arthroplasty Register (LROI) is a nationwide population-based register that includes information on joint arthroplasties in the Netherlands since 2007 (van Steenbergen et al. 2015). The LROI is initiated by the Netherlands Orthopaedic Association (NOV). Nearly all Dutch orthopedic surgeons are members of this society. The LROI is well supported by these members, resulting in a completeness of 99% of primary knee arthroplasties and 98% of knee revision arthroplasties and a high overall validity in 2016 (LROI 2017).

### Data collection

The LROI database contains information on patient, operation, and prosthesis characteristics (van Steenbergen et al. 2015). For each component a product number is registered. Prosthesis characteristics are derived from an implant library, which contains several core characteristics, including name, type (e.g., PS or CR TKA) and material of prostheses. These data are supplied by implant manufacturers or distributors in the Netherlands, resulting in a database containing almost 36,000 different implant components. If a product number is not present in the database when registered by a local hospital, the specific manufacturer is contacted to add their data to the implant library.

A primary knee arthroplasty is defined as the first implantation of a prosthesis. Knee revision arthroplasty is defined as any exchange (placement, replacement, or removal) of 1 or more components of the prosthesis. Revision procedures were categorized as minor if only the patella and insert were revised (excluding patella additions) and as major when at least the femur or tibia component was revised. Patella additions were studied as a separate category. Date of death of patients was obtained on a regular basis from Vektis, the national insurance database, which records vital status of Dutch citizens (Vektis 2017).

For the present study, we selected all 133,841 cemented fixed bearing primary TKAs for osteoarthritis in the period 2007–2016 with a PS or CR TKA system. Overall physical condition was scored using the ASA score (I–IV). The median follow-up was 4 years (0–10).

### Statistics

Survival time was calculated as the time between primary arthroplasty and first revision arthroplasty for any reason (Nelissen et al. 1992), death of the patient, or end of the study follow-up (January 1, 2017). 8-year all cause crude cumulative incidence of revision of CR and PS TKAs was calculated using competing risk analysis, where death was considered to be a competing risk (Andersen et al. 2012, Lacny et al. 2015, Wongworawat et al. 2015).

Multivariable Cox regression analyses were performed to compare the adjusted revision rates between the types of TKA system. Adjustments were made for age at surgery, sex, ASA score, and previous operations. Reasons for revision were compared between the type of TKA system (PS and CR TKA system) using a chi-square test. All statistical analyses were done using SPSS version 23.0 (IBM Corp, Armonk, NY, USA).

For the 95% confidence intervals (CI), we assumed that the number of observed cases followed a Poisson distribution.

## Funding and conflicts of interest

This research did not receive any grants.

None of the authors declare any competing interests.

## Results

### Patient characteristics

133,481 cemented fixed-bearing primary TKAs for osteoarthritis were registered by 100 hospitals in the period 2007–2016. Of these TKAs, 45% were a CR and 55% were a PS TKA. Patient characteristics were similar between CR and PS TKA systems (Table 1). A patellar component was placed during the primary procedure in 18% (n = 10,668) of CR TKAs and in 29% (n = 20,831) of PS TKAs. The proportion of CR TKAs per hospital ranged from 0% to 100%, with 20 hospitals using CR TKA systems in over 95% of all TKAs and 24 hospitals using PS TKA systems in over 95% of cases.

**Table 1.** Patient characteristics of patients who received a cemented fixed-bearing primary total knee arthroplasty for osteoarthritis with cruciate retaining (CR) or posterior stabilized (PS) knee system in the Netherlands in 2007–2016

	CR TKA	PS TKA	Total
	(n = 60,546)	(n = 73,295)	(n = 133,841)
Mean age (SD)	69 (9.3)	69 (9.4)	69 (9.3)
Age (%)			
< 50	2	2	2
50–59	14	15	15
60–69	36	36	36
70–79	35	35	35
≥ 80	13	12	12
Sex (%)			
Male	34	34	34
Female	66	66	66
ASA score (%)			
I	19	18	19
II	67	68	67
III–IV	14	14	14
Previous surgery^a^ (%)			
Yes	32	34	33
No	67	65	66
Unknown	1	1	1

a Includes meniscectomy, arthroscopy, osteotomy, osteosynthesis, ligament reconstruction, synovectomy, and other previous surgery.

TKA: total knee arthroplasty.

### Revision rates

**Figure 1.**
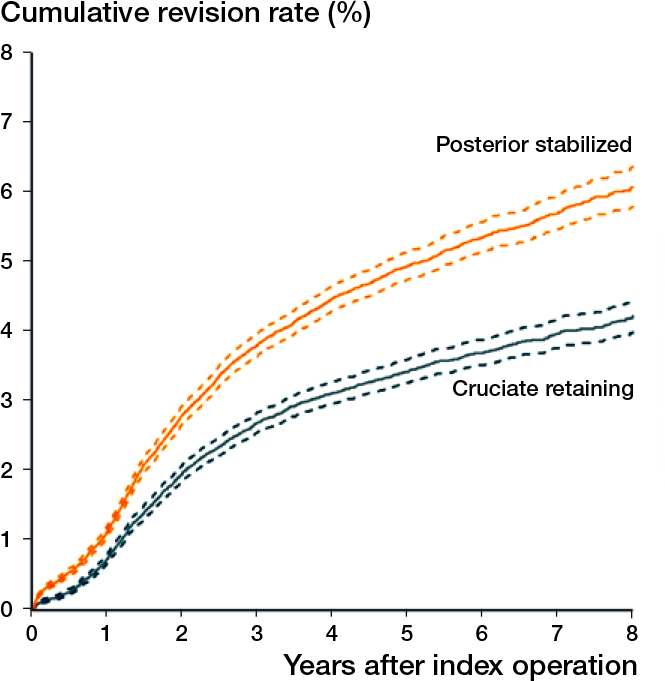
Cumulative revision rates of cemented cruciate retaining or posterior stabilized fixed bearing total knee arthroplasties for osteoarthritis by type of knee system in the Netherlands in 2007–2016 (n = 133,841). Dotted lines represent the 95% confidence interval.

Of all CR TKA systems, 4.2% (CI 4.0–4.4) were revised within 8 years, while 6.1% (CI 5.8–6.3) of PS TKAs were revised (Figure 1). Of all CR TKA systems 12.7% (CI 12.2–13.2) of the patients died and in 11.1% (CI 10.6–11.6) of primary PS TKAs the patients died within 8 years of the primary procedure.

Overall, the risk of a TKA being revised within 8 years after the primary procedure strongly depended on age, with TKAs in patients under 50 years of age being 2.4 (CI 2.1–2.8) times more likely to be revised, compared with TKAs in patients aged 60–69 years. TKA systems in patients aged over 80 years were half as likely to be revised (HR 0.5; CI 0.5–0.6). Furthermore, TKAs in patients who underwent a previous surgery to the same knee were 1.2 (CI 1.1–1.3) times more likely to be revised within 8 years (Table 2).

**Table 2.** Multivariate survival analyses of cemented cruciate retaining (CR) or posterior stabilized (PS) fixed bearing total knee arthroplasties for osteoarthritis in the Netherlands in 2007–2016

		Crude model		Adjusted model^a^	
	n	HRcrude	CI	HRadj	CI
TKA system					
CR (ref)	60,546	1.0		1.0	
PS	73,295	1.4^b^	1.4–1.5	1.5^b^	1.4–1.6
Age					
< 50	2,851	2.5^b^	2.2–2.9	2.4^b^	2.1–2.8
50–59	19,552	1.6^b^	1.4–1.7	1.4^b^	1.4–1.7
60–69 (ref)	47,982	1.0		1.0	
70–79	46,864	0.8^b^	0.7–0.9	0.8^b^	0.8–0.9
≥ 80	16,368	0.5^b^	0.4–0.6	0.5^b^	0.5–0.6
Sex					
Female (ref)	87,660	1.0		1.0	
Male	45,905	1.1^c^	1.0–1.1	1.0	0.9–1.1
ASA score					
I (ref)	24,298	1.0		1.0	
II	89,657	0.9^d^	0.9–1.0	1.1^c^	1.0–1.2
III–IV	18,164	1.0	0.9–1.1	1.3^b^	1.2–1.4
Previous operations^e^					
No (ref)	84,447	1.0		1.0	
Yes	42,503	1.4^b^	1.3–1.5	1.2^b^	1.1–1.3

TKA: total knee arthroplasty;

HR_crude_: crude hazard ratio; HR_adj_: adjusted hazard ratio.

a Adjusted for age at surgery, sex, ASA score, and previous operations.

b p < 0.001;

c p = 0.02;

d p = 0.04.

e Includes meniscectomy, arthroscopy, osteotomy, osteosynthesis, ligament reconstruction, synovectomy, and other previous surgery.

**Figure 2.**
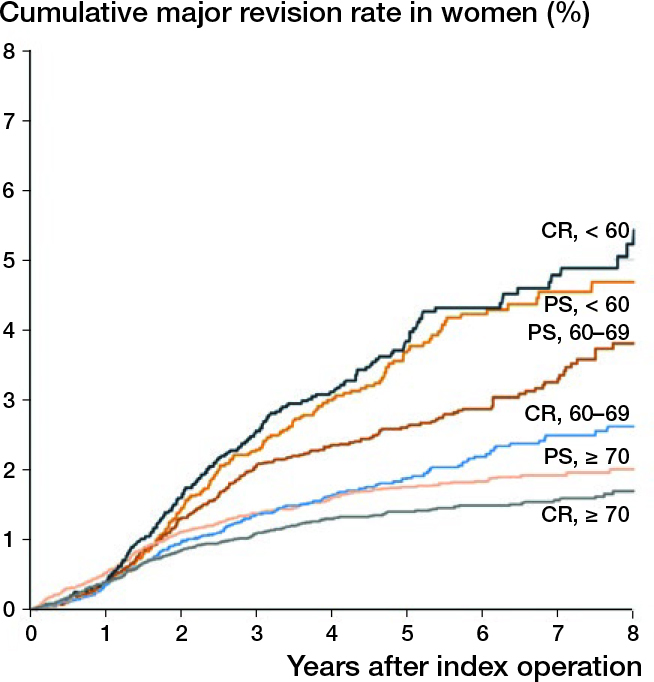
Cumulative revision rates of cemented cruciate retaining or posterior stabilized fixed bearing total knee arthroplasties for osteoarthritis by type of knee system per age category, stratified for sex, in the Netherlands in 2007–2016 (n = 133,841). CR: cruciate retaining TKA system; PS: posterior stabilized TKA system.

PS TKAs were 1.5 (CI 1.4–1.6) times more likely to be revised within 8 years, compared with CR TKA systems (Table 3). After excluding revisions for infection, a revision was 1.4 (CI 1.3–1.5) times more likely to be performed on a primary PS TKA. When stratified for sex and age category, the difference in cumulative revision rates was predominant for younger males. Of PS TKA systems in males under 60 years of age, 13% (CI 11–15) were revised within 8 years of the primary procedure, compared with 7.2% (CI 6.1–8.5) of CR TKA systems. This difference was not present in younger females (CR 8.2% (CI 7.2–9.3); PS 9.4% (CI 8.5–11)) (Figure 2, see Supplementary data). Regarding general health (ASA score), the difference in 8-year cumulative revision between CR and PS TKA systems was most prominent in patients with ASA score II (Table 4, see Supplementary data).

**Table 3.** Multivariate survival analyses of cemented fixed bearing primary cruciate retaining (CR) or posterior stabilized (PS) total knee arthroplasty for osteoarthritis in the Netherlands in 2007–2016

	TKA	Primary	Revisions	Crude model		Adjusted model^a^	
	system	TKAs (n)	(n)	HR_crude_	CI	HR_adj_	CI
Any type of revision							
CR (ref)	60,546	1,718	1.0		1.0		
PS	73,295	2,659	1.4^b^	1.4–1.5	1.5^b^	1.4–1.6	
Minor revisions^c^							
CR (ref)	60,546	333	1.0		1.0		
PS	73,295	713	2.0^b^	1.7–2.2	2.0^b^	1.7–2.3	
Patella addition^d^							
CR (ref)	60,546	402	1.0		1.0		
PS	73,295	555	1.3^b^	1.1–1.5	1.3^b^	1.1–1.5	
Major revisions^e^							
CR (ref)	60,546	911	1.0		1.0		
PS	73,295	1,238	1.3^b^	1.2–1.4	1.3^b^	1.2–1.4	

For abbreviations, see Table 2

a Adjusted for age at surgery, sex, ASA score, and previous operations.

b p < 0.001.

c Only insert and/or patella exchange (excluding patella addition).

d Only patella addition. A patellar component was placed during the primary procedure of 17.7% (n = 10,668) of cruciate retaining TKAs and of 28.5% (n = 20,831) of posterior stabilized TKAs.

e Revision of at least femur or tibia.

**Table 4.** Cumulative 8-year revision rates of cemented fixed bearing total knee arthroplasties for osteoarthritis with cruciate retaining (CR) or posterior stabilized (PS) knee system, stratified for sex, age, and ASA score, in the Netherlands in 2007–2016 (n = 133,841)

	CR TKA system		PS TKA system	
Sex, age	8-year revision		8-year revision	
ASA-score	n	rate (%) (CI)	n	rate (%) (CI)
**Male, < 60 years**				
ASA I	1,561	7.2 (5.6–9.2)^a^	1,753	12.1 (9.8–15.0)^a^
ASA II	2,105	7.8 (6.0–10.3)^a^	2,605	14.4 (11.4–18.1)^a^
ASA III–IV	236	6.1 (3.3–11.3)	347	13.0 (7.7–22.2)
Total	4,015	7.2 (6.1–8.5)^a^	4,842	12.7 (11.1–14.5)^a^
**Male, 60–69 years**				
ASA I	1,975	2.7 (2.0–3.7)^a^	2,161	5.6 (4.4–7.2)^a^
ASA II	5,127	4.3 (3.5–5.2)^a^	6,408	6.5 (5.6–7.7)^a^
ASA III–IV	890	4.9 (3.3–7.5)	1,057	7.6 (5.5–10.6)
Total	8,193	4.0 (3.4–4.7)^a^	9,835	6.3 (5.6–7.1)^a^
**Male, ≥ 70 years**				
ASA I	1,091	3.4 (2.0–5.6)	1,310	5.5 (4.1–7.5)
ASA II	5,766	2.3 (1.9–2.9)	6,765	3.4 (2.8–4.2)
ASA III–IV	1,546	2.2 (1.5–3.4)	2,034	4.0 (2.9–5.5)
Total	8,621	2.6 (2.2–3.2)^a^	10,365	4.0 (3.4–4.6)^a^
**Female, < 60 years**				
ASA I	1,717	8.3 (6.7–10.4)	2,124	8.7 (7.1–10.7)
ASA II	3,444	7.5 (6.3–8.9)	4,579	9.6 (8.3–11.0)
ASA III–IV	533	8.9 (4.4–18.1)	728	11.9 (8.7–16.4)
Total	5,876	8.2 (7.2–9.3)	7,648	9.4 (8.5–10.5)
**Female, 60–69 years**				
ASA I	2,690	5.7 (4.6–7.2)	3,070	5.4 (4.2–7.0)
ASA II	9,030	4.0 (3.5–4.7)^a^	11,011	7.2 (6.2–8.3)^a^
ASA III–IV	1,410	5.9 (4.3–8.1)	1,916	7.9 (6.0–10.5)
Total	13,499	4.7 (4.2–5.3)	16,400	6.6 (6.0–7.3)
**Female, ≥ 70 years**				
ASA I	2,259	2.0 (1.4–2.8)^a^	2,524	4.4 (3.5–5.6)^a^
ASA II	14,016	3.0 (2.7–3.5)^a^	16,562	4.0 (3.6–4.5)^a^
ASA III–IV	3,280	3.1 (2.4–4.0)	4,120	3.8 (3.0–4.7)
Total	20,187	2.9 (2.6–3.3)^a^	23,984	4.0 (3.6–4.3)^a^

TKA: total knee arthroplasty.

a p < 0.05 for PS compared with CR TKAs.

## Revision characteristics

Of all primary cemented fixed-bearing CR or PS TKAs for osteoarthritis in the period 2007–2016, 4,377 (CR 1,718; PS 2,659) TKAs were revised within 8 years of the primary procedure. Of these revisions, 49% were major (CR 53%; PS 47%) and 24% were minor revisions (CR 19%; PS 27%). Furthermore, 22% of all revisions were a patella addition (CR 23%; PS 21%).

A minor revision was 2.0 (CI 1.7–2.3) times more likely to be performed on a primary PS TKA, compared with a primary CR TKA within 8 years of the primary procedure. After excluding revisions for infection, a minor revision was 1.8 (CI 1.5–2.1) times more likely to be performed on a primary PS TKA. A major revision was 1.3 (CI 1.2–1.4) times more likely to be performed on a PS TKA system compared with a CR TKA system (Table 3).

**Figure 3.**
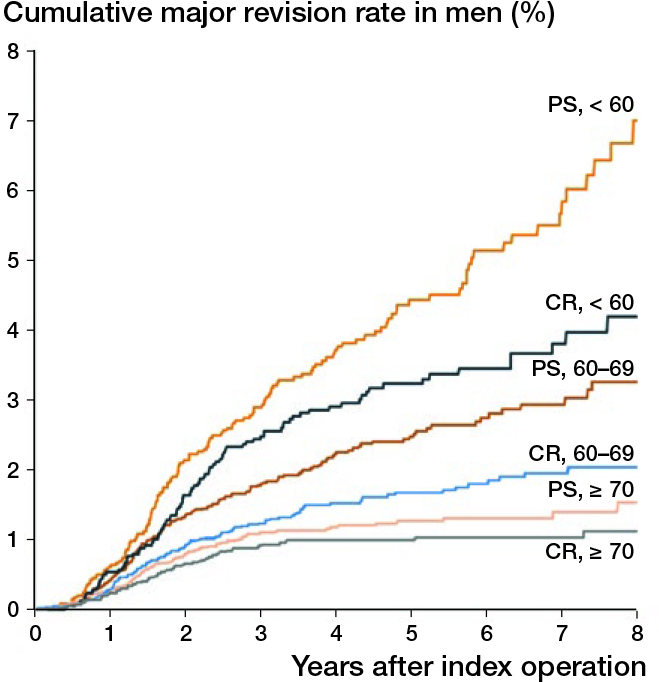
Cumulative major revision rates of cemented cruciate retaining or posterior stabilized fixed bearing total knee arthroplasties for osteoarthritis by type of knee system per age category, stratified for sex, in the Netherlands in 2007–2016 (n = 133,841). CR: cruciate retaining TKA system; PS: posterior stabilized TKA system

When zooming in on patient characteristics, in general, CR TKAs had higher revision rates for female patients compared with male patients (Table 5, see Supplementary data). Male patients who received a PS TKA system significantly more often underwent a minor revision procedure (2.0% CI 1.7–2.3) compared with male patients who received a CR TKA system (1.1% CI 0.9–1.3). These results were predominant for younger males (PS TKAs 3.7% (CI 3.0–4.7); CR TKAs 1.8% (CI 1.4–2.5)). In females, minor revision rates between CR and PS TKA systems also differed for younger patients (PS TKAs:= 2.7% (CI 2.1–3.4); CR TKAs:= 1.2% (CI 0.9–1.6)). This difference was less prominent. Furthermore, major revisions were more common in younger males who received a PS TKA system (7.0% CI 5.6–8.7), compared with younger males who received a CR TKA system (4.2% CI 3.3–5.3; Table 5, see Supplementary data). The differences in revision rates between CR and PS TKA systems increased as the number of years after the primary TKA increased. In females, major revision rates between CR and PS TKA systems did not differ for patients under 60 years of age (Figure 3).

**Table 5.** Cumulative minor and major 8-year revision rates of cemented fixed bearing total knee arthroplasties for osteoarthritis by type of TKA system, stratified for sex and age, in the Netherlands in 2007–2016 (n = 133,841)

Type of revision, sex	CR TKA system 8-year revision		PS TKA system 8-year revision	
Age, years	n	rate (%) (CI)	n	rate (%) (CI)
**Minor** (only insert and/or patella exchange^a^)				
**Male**				
< 60	4,015	1.8 (1.4–2.5)^b^	4,842	3.7 (3.0–4.7)^b^
60–69	8,193	1.1 (0.8–1.5)	9,835	1.8 (1.4–2.2)
≥ 70	8,624	0.7 (0.4–1.2)	10,365	1.3 (1.0–1.7)
Total	20,844	1.1 (0.9–1.3)^b^	25,061	2.0 (1.7–2.3)^b^
**Female**				
< 60	5,876	1.2 (0.9–1.6)^b^	7,648	2.7 (2.1–3.4)^b^
60–69	13,499	0.8 (0.6–1.1)	16,400	1.2 (0.9–1.5)
≥ 70	20,187	0.4 (0.3–0.6)^b^	23,984	0.8 (0.7–1.0)^b^
Total	39,596	0.7 (0.6–0.8)^b^	48,064	1.3 (1.1–1.4)^b^
**Major** (including femur or tibia)				
**Male**				
< 60	4,015	4.2 (3.3–5.3)^b^	4,842	7.0 (5.6–8.7)^b^
60–69	8,193	2.0 (1.6–2.5)^b^	9,835	3.3 (2.7–3.9)^b^
≥ 70	8,624	1.1 (0.9–1.5)	10,365	1.5 (1.2–2.0)
Total	20,844	2.1 (1.8–2.4)^b^	25,061	3.4 (3.0–3.8)^b^
**Female**				
< 60	5,876	5.2 (4.4–6.2)	7,648	4.7 (4.0–5.5)
60–69	13,499	2.6 (2.2–3.1)^b^	16,400	3.8 (3.3–4.4)^b^
≥ 70	20,187	1.7 (1.5–2.0)	23,984	2.0 (1.8–2.3)
Total	39,596	2.5 (2.3–2.7)^b^	48,064	3.0 (2.8–3.3)^b^

a Excluding patella addition.

b p < 0.05 for PS compared with CR TKAs.

### Reasons for revision

Patellar pain was the most common reason for revision within 8 years for both CR and PS TKA systems. Reasons for revision were different for minor compared with major revisions. The most common reason for performing a minor revision was instability (CR 41%; PS 37%), followed by infection, which was more often the reason for minor revisions in PS TKAs, compared with CR TKAs. Loosening of the tibial component was the most common reason for performing a major revision, though it was more common on a PS (41%) compared with a CR TKA system (27%; p < 0.001). Furthermore, instability was more often a reason for performing a major revision on a CR (36%) compared with a PS TKA system (23%; p < 0.001) (Table 6, see Supplementary data).

**Table 6.** Reasons for minor or major revisions of cruciate retaining and posterior stabilized cemented fixed bearing TKA systems for osteoarthritis in the Netherlands in 2007–2016

	Minor revision^a^ (n = 1,046)		Major revision^b^ (n = 2,149)		Any revision^c^ (n = 4,377)	
	CR (n = 333) PS (n = 713)		CR (n = 911) PS (n = 1,238)		CR (n = 1,718) PS (n = 2,659)	
Reason for revision	n (%)	n (%)	n (%)	n (%)	n (%)	n (%)
Patellar pain	102 (30.6)	18 (26.2)	84 (9.2)	145 (11.7)	549 (32.0)	842 (31.7)
Instability	138 (41.4)	263 (36.9)	327 (35.9)	284 (22.9)^g^, ^h^	477 (27.8)	567 (21.3)^g^, ^h^
Loosening tibial component	0 (0.0)	3 (0.4)	242 (26.6)	505 (40.8)^g^, ^h^	243 (14.1)	513 (19.3)^g^, ^h^
Infection	67 (20.1)	207 (29.0)^f^	161 (17.7)	252 (20.4)	244 (14.2)	499 (18.8)^g^, ^h^
Malalignment	6 (1.8)	11 (1.5)	254 (27.9)	371 (30.0)	265 (15.4)	387 (14.6)
Loosening femoral component	1 (0.3)	1 (0.1)	78 (8.6)	117 (9.5)	80 (4.7)	119 (4.5)
Arthrofibrosis	20 (6.0)	30 (4.2)	32 (3.5)	55 (4.4)	61 (3.6)	93 (3.5)
Revision after removal	0 (0.0)	1 (0.1)	48 (5.3)	97 (7.8)^e^	48 (2.8)	98 (3.7)
Patellar dislocation	10 (3.0)	15 (2.1)	37 (4.1)	30 (2.4)^e^	74 (4.3)	71 (2.7)^e^
Wear of inlay	18 (5.4)	33 (4.6)	18 (2.0)	25 (2.0)	37 (2.2)	61 (2.3)
Periprosthetic fracture	0 (0.0)	6 (0.8)	21 (2.3)	69 (5.6)^f^	21 (1.2)	75 (2.8)^f^
Loosening patellar component	10 (3.0)	14 (2.0)	6 (0.7)	9 (0.7)	20 (1.2)	31 (1.2)
Other reason for revision	53 (15.6)	118 (15.3)	112 (12.3)	122 (9.9)^d^	219 (12.7)	315 (11.8)

a Only insert and/or patella exchange (excluding patella addition).

b Revision of at least femur or tibia.

c Any type of revision, including patella addition (CR n = 402; PS n = 555). A patellar component was placed during the primary proce-dure of 17.7% (n = 10,668) of cruciate retaining TKAs and of 28.5% (n = 20.831) of posterior stabilized TKAs.

d p = 0.03

e p = 0.01

f p = 0.001

g p < 0.001

h Categories differ significantly for PS compared with CR TKAs after applying a Bonferroni correction (p = 0.05/39 = 0.001).

## Discussion

### Main findings and previous research

We found higher mid-term minor and major revision rates in patients with PS TKA systems, especially in males younger than 60 years. These results are not in line with several previous review studies comparing CR and PS TKA designs (Jacobs et al. 2005, Verra et al. 2015, Jasper et al. 2016). Possible reasons for this difference might be non-consecutive study series with subsequent selection bias, smaller study sizes, and patients lost to follow-up in these previous studies, emphasizing the importance of analyzing population-based data. However, our results confirm results of other previous studies (Abdel et al. 2011, Vertullo et al. 2017).

As life expectancy is growing and the mean age of patients undergoing primary arthroplasty continues to decrease, revision rate and survival of implants are becoming increasingly important (Carr et al. 2012, Pabinger et al. 2013, Hamilton et al. 2015, Shah et al. 2017). The number of patients currently living with a TKA suggests a large potential revision healthcare burden (Hamilton et al. 2015). International literature has shown that revision rates differ for a number of patient characteristics, including age, sex, general health, physical activity, BMI, and smoking (McCalden et al. 2013, Pabinger et al. 2013, Apold et al. 2014, Jasper et al. 2016, Kunutsor et al. 2016, LROI 2017).

Preoperative decision-making is a complex process for both the surgeon and patient (Carr et al. 2012). In particular age and weight are important indicators for surgery. Patients aged less than 55 years or with preoperative morbid obesity have more variable outcomes after knee replacement than those older than 55 years and those with a lower BMI (Carr et al. 2012). A recent study showed that patients aged under 60 years had an increased risk for revision of their TKA, with time to revision reaching a peak around 5 years after implantation (Bayliss et al. 2017). Grade of preoperative radiographic destruction in combination with high age have been reported as important factors for preoperative decision-making (Verra et al. 2015). However, patient characteristics do not appear to be the main motive when choosing between a CR and PS TKA system in the Netherlands, since large variation is seen between hospitals. In patients with a functional PCL, the decision on which design should be used depends largely on the preference and training of the surgeon (Jacobs et al. 2005, van den Boom et al. 2009). Our study shows, however, that mid-term revision rates are higher for PS TKA systems, especially for younger males. This implies a need to consider these patient characteristics when choosing the type of TKA system.

The incidence of TKA is increasing (LROI 2017), and is projected to increase rapidly in the near future (Carr et al. 2012), particularly in young patients (Kurtz et al. 2009). As the number of primary TKAs grows, the number of revisions is expected to increase as well (Ethgen et al. 2004, Hamilton et al. 2015). Although overall improvement is seen in patients’ health and function, outcome of knee revision arthroplasty is worse than that of the primary procedure, with even worse results for early revisions in young patients (Hardeman et al. 2012). Survival rates of revision surgery are consistently reported at around 80% at 10 years (Hardeman et al. 2012, Hamilton et al. 2015). Younger patients are also more likely to undergo a reoperation, without revision of TKA system components (Zmistowski et al. 2011).

Large discrepancies exist between the extensiveness of revision arthroplasties. The difference in mid-term revision rates between CR and PS TKA systems was more prominent for minor revisions (i.e., insert or patella revision). This may partially be a consequence of hospital policy or surgeon preference. This does not, however, explain the sex difference. For either minor or major revisions instability was a frequent reason for revision, indicating an important motivation for deciding on the preference for CR and PS TKA systems. The higher frequency of loosening of the tibial component as a reason for major revision in PS TKAs, compared with CR TKAs, might possibly be the result of higher shear forces on the tibial component in the PS TKA models. Moreover, after removing the PCL the flexion gap is usually bigger and inserts tend to be slightly thicker in PS models than in CR models, which may affect shear forces. However, these are only speculations as an observational study cannot disclose causative mechanisms. The higher frequency of infection as reason for revision in PS TKA systems compared with CR TKA systems was unexpected. Although types of revisions are considered to be small, these findings are of importance for the results of TKAs in terms of the risk for complications, the patient’s perspective on the results, and health costs.

### Strengths and limitations

Using a generic matrix on design features across all implants enables analyses of conceptual design issues of implants. With the help of the implant library, the register shows which component is registered for every reference number. This reduces the risk of registration errors. Furthermore, the LROI contains a large population-based nationwide database of primary TKAs, with a completeness of nearly 100% (van Steenbergen et al. 2015, LROI 2016). This is the first study on this subject with this many included procedures in the known literature. Registry studies like this study have the advantage of analyzing population-based data, avoiding selection bias, and minimizing misclassification error. Nevertheless, registry data also have their drawbacks since only limited variables are registered and causality cannot be proven due to its observational nature. Data from registries should accompany prospective cohort studies like randomized controlled trials, which can collect a large number of variables. An analysis of patient characteristics determining the choice for a CR or PS TKA design would be interesting, but also difficult, if not impossible, to undertake. The latter stresses even more the importance of our conclusions: which TKA, CR or PS, is appropriate for a typical patient.

A limitation of this study is that there is probably residual confounding due to BMI, Charnley score, and smoking. BMI is known to be associated with TKA revision and mortality rates (Carr et al. 2012, Hamilton et al. 2015). The same is true for the degree of osteoarthritis in lower extremity joints (i.e., Charnley score) and smoking. These confounding factors have been recorded only since July 2013 and could therefore not be included in the present study. Furthermore, a TKA has proven to be a successful procedure, resulting in a small proportion of revision procedures. Despite the large database of primary TKAs, more data are needed to study characteristics of these revision procedures in more detail. This study was unable to study patient characteristics sufficiently in relation to reasons for minor or major revisions by type of TKA system, particularly in younger patients. Infections are only registered in the LROI when 1 of the prosthesis components is revised. Furthermore, only (suspected) prosthetic joint infections as reason for revision were registered, since this is registered at the time of operation and not based on microbiology results. As had been shown earlier, implant registries largely underscore prosthetic joint infections (Gundtoft et al. 2015). When excluding revisions where infection was registered to be a reason for revision, however, revision rates were still significantly higher for PS TKAs, compared with CR TKAs.

Further research is recommended on the effect of revision procedures on the patient’s perspective on the results. Patient-reported outcome measures are important in the evaluation of revisions, since an important indication is pain. Data on pain in the postoperative period were not available for our study population (LROI 2017). Furthermore, research is warranted on high-flexion TKAs as a factor influencing revision rates. This prosthesis characteristic could not currently be detected. Finally, only 1 prosthesis characteristic was studied, while a complex interaction between factors has become increasingly evident. An understanding of this interplay between prosthesis characteristics on the outcome of TKA survival is needed to assist surgeons to optimize outcomes for patients (Carr et al. 2012).

### Summary

Revision rates within 8 years of the primary procedure were 1.5 times higher for PS TKA compared with CR TKA systems. This was more prominent in male patients under 60 years of age. Although this effect was largest in minor revisions, it was also found for major revisions. Our data may give guidance for surgical decision-making for specific patient groups when choosing a CR or PS TKA system.

### Supplementary data

Figure 2 and Tables 4–6 are available as supplementary data in the online version of this article, http://dx.doi.org/ 10.1080/17453674.2018.1518570

The authors contributed to: (1) study design and study protocol, (2) gathered data, (3) analyzed data, (4) initial draft, and (5) final draft. AS and LS contributed to: 1–5; GD, BS, and RP contributed to: 2, 4, 5; RN contributed to: 1, 2, 4, 5.

*Acta* thanks Asgeir Gudnason and Mika Niemeläinen for help with peer review of this study.

## Supplementary Material

Supplemental Material
