# Supplementary material for: Higher mid-term revision rates of posterior stabilized compared with cruciate retaining total knee arthroplasties: 133,841 cemented arthroplasties for osteoarthritis in the Netherlands in 2007–2016
Source: Acta Orthop. 2018 Oct 23;89(6):640–5. doi: 10.1080/17453674.2018.1518570 (PMC6300738; doi:10.1080/17453674.2018.1518570)
Supplement: Supplemental Material [file IORT_A_1518570_SM3055.pdf]

Supplementary data

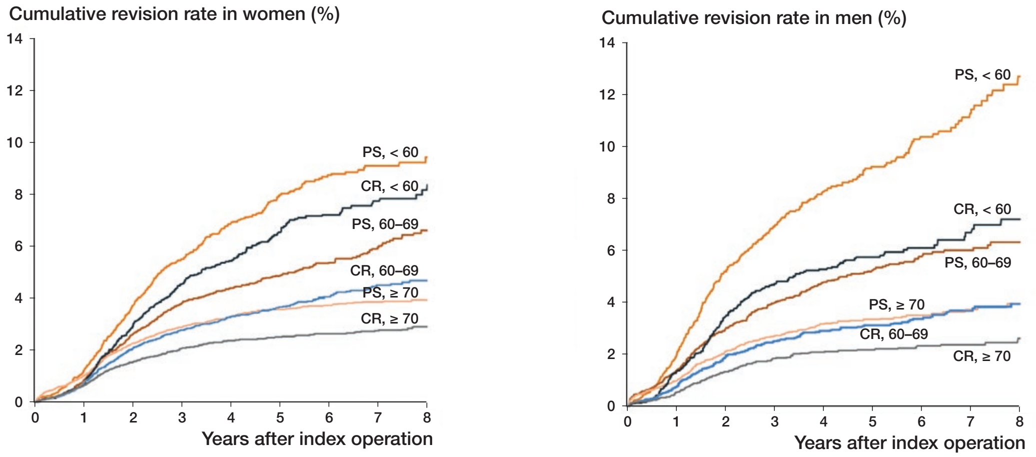

Figure 2. Cumulative revision rates of cemented cruciate retaining or posterior stabilized fixed bearing total knee arthroplasties for osteoarthritis by type of knee system per age category, stratified for sex, in the Netherlands in 2007–2016 (n = 133,841). CR: cruciate retaining TKA system; PS: posterior stabilized TKA system.

Table 4. Cumulative 8-year revision rates of cemented fixed bearing total knee arthroplasties for osteoarthritis with cruciate retaining (CR) or posterior stabilized (PS) knee system, stratified for sex, age, and ASA score, in the Netherlands in 2007–2016 (n = 133,841)

Sex, age<br>ASA-score	CR TKA system<br>n	 8-year revision rate (%) (CI)	PS TKA system<br>n	 8-year revision rate (%) (CI)
**Male, < 60 years**				
ASA I	1,561	7.2 (5.6–9.2) [a]	1,753	12.1 (9.8–15.0) [a]
ASA II	2,105	7.8 (6.0–10.3) [a]	2,605	14.4 (11.4–18.1) [a]
ASA III–IV	236	6.1 (3.3–11.3)	347	13.0 (7.7–22.2)
Total	4,015	7.2 (6.1–8.5) [a]	4,842	12.7 (11.1–14.5) [a]
**Male, 60–69 years**				
ASA I	1,975	2.7 (2.0–3.7) [a]	2,161	5.6 (4.4–7.2) [a]
ASA II	5,127	4.3 (3.5–5.2) [a]	6,408	6.5 (5.6–7.7) [a]
ASA III–IV	890	4.9 (3.3–7.5)	1,057	7.6 (5.5–10.6)
Total	8,193	4.0 (3.4–4.7) [a]	9,835	6.3 (5.6–7.1) [a]
**Male, ≥ 70 years**				
ASA I	1,091	3.4 (2.0–5.6)	1,310	5.5 (4.1–7.5)
ASA II	5,766	2.3 (1.9–2.9)	6,765	3.4 (2.8–4.2)
ASA III–IV	1,546	2.2 (1.5–3.4)	2,034	4.0 (2.9–5.5)
Total	8,621	2.6 (2.2–3.2) [a]	10,365	4.0 (3.4–4.6) [a]
**Female, < 60 years**				
ASA I	1,717	8.3 (6.7–10.4)	2,124	8.7 (7.1–10.7)
ASA II	3,444	7.5 (6.3–8.9)	4,579	9.6 (8.3–11.0)
ASA III–IV	533	8.9 (4.4–18.1)	728	11.9 (8.7–16.4)
Total	5,876	8.2 (7.2–9.3)	7,648	9.4 (8.5–10.5)
**Female, 60–69 years**				
ASA I	2,690	5.7 (4.6–7.2)	3,070	5.4 (4.2–7.0)
ASA II	9,030	4.0 (3.5–4.7) [a]	11,011	7.2 (6.2–8.3) [a]
ASA III–IV	1,410	5.9 (4.3–8.1)	1,916	7.9 (6.0–10.5)
Total	13,499	4.7 (4.2–5.3)	16,400	6.6 (6.0–7.3)
**Female, ≥ 70 years**				
ASA I	2,259	2.0 (1.4–2.8) [a]	2,524	4.4 (3.5–5.6) [a]
ASA II	14,016	3.0 (2.7–3.5) [a]	16,562	4.0 (3.6–4.5) [a]
ASA III–IV	3,280	3.1 (2.4–4.0)	4,120	3.8 (3.0–4.7)
Total	20,187	2.9 (2.6–3.3) [a]	23,984	4.0 (3.6–4.3) [a]

TKA: total knee arthroplasty.
[a] p < 0.05 for PS compared with CR TKAs.

Table 5. Cumulative minor and major 8-year revision rates of cemented fixed bearing total knee arthroplasties for osteoarthritis by type of TKA system, stratified for sex and age, in the Netherlands in 2007–2016 (n = 133,841)

Type of revision, sex<br>Age, years	CR TKA system<br>n	 8-year revision rate (%) (CI)	PS TKA system<br>n	 8-year revision rate (%) (CI)
**Minor** (only insert and/or patella exchange [a])				
**Male**				
< 60	4,015	1.8 (1.4–2.5) [b]	4,842	3.7 (3.0–4.7) [b]
60–69	8,193	1.1 (0.8–1.5)	9,835	1.8 (1.4–2.2)
≥ 70	8,624	0.7 (0.4–1.2)	10,365	1.3 (1.0–1.7)
Total	20,844	1.1 (0.9–1.3) [b]	25,061	2.0 (1.7–2.3) [b]
**Female**				
< 60	5,876	1.2 (0.9–1.6) [b]	7,648	2.7 (2.1–3.4) [b]
60–69	13,499	0.8 (0.6–1.1)	16,400	1.2 (0.9–1.5)
≥ 70	20,187	0.4 (0.3–0.6) [b]	23,984	0.8 (0.7–1.0) [b]
Total	39,596	0.7 (0.6–0.8) [b]	48,064	1.3 (1.1–1.4) [b]
**Major** (including femur or tibia)				
**Male**				
< 60	4,015	4.2 (3.3–5.3) [b]	4,842	7.0 (5.6–8.7) [b]
60–69	8,193	2.0 (1.6–2.5) [b]	9,835	3.3 (2.7–3.9) [b]
≥ 70	8,624	1.1 (0.9–1.5)	10,365	1.5 (1.2–2.0)
Total	20,844	2.1 (1.8–2.4) [b]	25,061	3.4 (3.0–3.8) [b]
**Female**				
< 60	5,876	5.2 (4.4–6.2)	7,648	4.7 (4.0–5.5)
60–69	13,499	2.6 (2.2–3.1) [b]	16,400	3.8 (3.3–4.4) [b]
≥ 70	20,187	1.7 (1.5–2.0)	23,984	2.0 (1.8–2.3)
Total	39,596	2.5 (2.3–2.7) [b]	48,064	3.0 (2.8–3.3) [b]

[a] Excluding patella addition.
[b] p < 0.05 for PS compared with CR TKAs.

Table 6. Reasons for minor or major revisions of cruciate retaining and posterior stabilized cemented fixed bearing TKA systems for osteoarthritis in the Netherlands in 2007–2016

	Minor revision [a] (n = 1,046)		Major revision [b] (n = 2,149)		Any revision [c] (n = 4,377)	
	CR (n = 333)	PS (n = 713)	CR (n = 911)	PS (n = 1,238)	CR (n = 1,718)	PS (n = 2,659)
Reason for revision	n (%)	n (%)	n (%)	n (%)	n (%)	n (%)
Patellar pain	102 (30.6)	18 (26.2)	84 (9.2)	145 (11.7)	549 (32.0)	842 (31.7)
Instability	138 (41.4)	263 (36.9)	327 (35.9)	284 (22.9) [g, h]	477 (27.8)	567 (21.3) [g, h]
Loosening tibial component	0 (0.0)	3 (0.4)	242 (26.6)	505 (40.8) [g, h]	243 (14.1)	513 (19.3) [g, h]
Infection	67 (20.1)	207 (29.0) [f]	161 (17.7)	252 (20.4)	244 (14.2)	499 (18.8) [g, h]
Malalignment	6 (1.8)	11 (1.5)	254 (27.9)	371 (30.0)	265 (15.4)	387 (14.6)
Loosening femoral component	1 (0.3)	1 (0.1)	78 (8.6)	117 (9.5)	80 (4.7)	119 (4.5)
Arthrofibrosis	20 (6.0)	30 (4.2)	32 (3.5)	55 (4.4)	61 (3.6)	93 (3.5)
Revision after removal	0 (0.0)	1 (0.1)	48 (5.3)	97 (7.8) [e]	48 (2.8)	98 (3.7)
Patellar dislocation	10 (3.0)	15 (2.1)	37 (4.1)	30 (2.4) [e]	74 (4.3)	71 (2.7) [e]
Wear of inlay	18 (5.4)	33 (4.6)	18 (2.0)	25 (2.0)	37 (2.2)	61 (2.3)
Periprosthetic fracture	0 (0.0)	6 (0.8)	21 (2.3)	69 (5.6) [f]	21 (1.2)	75 (2.8) [f]
Loosening patellar component	10 (3.0)	14 (2.0)	6 (0.7)	9 (0.7)	20 (1.2)	31 (1.2)
Other reason for revision	53 (15.6)	118 (15.3)	112 (12.3)	122 (9.9) [d]	219 (12.7)	315 (11.8)

[a] Only insert and/or patella exchange (excluding patella addition).
[b] Revision of at least femur or tibia.
[c] Any type of revision, including patella addition (CR n = 402; PS n = 555). A patellar component was placed during the primary procedure of 17.7% (n = 10,668) of cruciate retaining TKAs and of 28.5% (n = 20.831) of posterior stabilized TKAs.
[d] $p = 0.03$
[e] $p = 0.01$
[f] $p = 0.001$
[g] $p < 0.001$
[h] Categories differ significantly for PS compared with CR TKAs after applying a Bonferroni correction ($p = 0.05/39=0.001$).
